# A Conceptual Framework for Blockchain Enhanced Information Modeling for Healing and Therapeutic Design

**DOI:** 10.3390/ijerph19138218

**Published:** 2022-07-05

**Authors:** Zhen Liu, Zulan Yang, Mingjie Liang, Yi Liu, Mohamed Osmani, Peter Demian

**Affiliations:** 1 School of Design, South China University of Technology, Guangzhou 510006, China; liuzjames@scut.edu.cn; 2 School of Innovation, Guangzhou Academy of Fine Arts, No. 257 Changgang Road, Guangzhou 510261, China; liuyi@gzarts.edu.cn; 3 School of Architecture, Building and Civil Engineering, Loughborough University, Loughborough LE11 3TU, UK; m.osmani@lboro.ac.uk (M.O.); p.demian@lboro.ac.uk (P.D.)

**Keywords:** healing and therapeutic design, blockchain, building information modeling (BIM), landscape information modeling (LIM), city information modeling (CIM), art therapy, mental health, sustainable development, non-fungible token (NFT), Health Metaverse

## Abstract

In the face of the health challenges caused by the COVID-19 pandemic, healing and therapeutic design (HTD) as interventions can help with improving people’s health. It is considered to have great potential to promote health in the forms of art, architecture, landscape, space, and environment. However, there are insufficient design approaches to address the challenges during the HTD process. An increased number of studies have shown that emerging information modeling (IM) such as building information modeling (BIM), landscape information modeling (LIM), and city information modeling (CIM) coupled with blockchain (BC) functionalities have the potential to enhance designers’ HTD by considering important design elements, namely design variables, design knowledge, and design decision. It can also address challenges during the design process, such as design changes, conflicts in design requirements, the lack of design evaluation tools and frameworks, and incomplete design information. Therefore, this paper aims to develop a conceptual BC enhanced IM for HTD (BC-HTD) framework that addresses the challenges in the HTD and promotes health and well-being. The structure of BC-HTD framework is twofold: (1) a conceptual high-level framework comprising three levels: user; system; and information, (2) a conceptual low-level framework of detailed content at the system level, which has been constructed using a mixed quantitative and qualitative method of literature analysis, and validated via a pre-interview questionnaire survey and follow-up interviews with industry experts and academics. This paper analyzes the process of BC enhanced HTD and the knowledge management of HTD to aid design decisions in managing design information. This paper is the first attempt to apply the advantages of BC enabled IM to enhance the HTD process. The results of this study can foster and propel new research pathways and knowledge on the value of design in the form of non-fungible token (NFT) based on the extended advantages of BC in the field of design, which can fully mobilize the healing and therapeutic behaviors of designers and the advantage potential of HTD to promote health, and realize the vision of Health Metaverse in the context of sustainable development.

## 1. Introduction

The coronavirus disease 2019 (COVID-19) has triggered a global mental health crisis [1]. Recent studies reported that several population groups, such as children [2], the elderly [3], caregivers [4], and medical staff [5], have suffered various mental health issues, including mood disorder, depression, anxiety, and stress. In the face of the impact of the COVID-19 epidemic, the World Health Organization has put forward a series of recommendations for epidemic prevention, such as home isolation, social distancing, and wearing masks [6]. However, prolonged isolation measures have had significant psychological consequences [7]. Amerio et al. point out that the built environment of housing can affect people’s mental health when staying home for a long period [8]. Moreover, environmental factors such as landscape quality and planting in the home space also affect people’s mental health [9]. Therefore, it is critical to devise measures to deal with the health threat brought by the COVID-19 to various groups.

Facing the health challenges posed by the COVID-19 pandemic, applications of healing and therapeutic design (HTD) in the field of art, architecture, landscape, space, and environment to overcome health barriers through the enhancement of aesthetics in design process have shown a positive effect on promoting health [10]. Moreover, the process of HTD based on information modeling (IM) can make better use of design information, strengthen design communication, and promote healing behavior [11]. In addition, faced with the problem of data privacy of patients under the COVID-19, blockchain (BC) has the advantage of protecting data privacy in the field of health care [12]. However, although HTD can help with improving people’s health, there are design challenges in the whole process of HTD, such as changes and conflicts in design requirements [13], and incomplete consideration of design features [14,15]. Furthermore, BC and IM pay little attention to the application of HTD aiming at promoting health and addressing the challenges of HTD more generally. Therefore, this paper aims to explore the potential positive effects of integrating both HTD and the BC enhanced IM, and develop a conceptual BC enhanced IM for HTD (BC-HTD) framework that provides application value in the context of the COVID-19 pandemic.

## 2. Healing and Therapeutic Design (HTD) Potential for Addressing Health Problems of COVID-19

A variety of non-drug treatments, art, and design have shown a positive role in promoting mental health [16]. Art therapy is a non-verbal therapeutic approach [17] that promotes health by expressing and communicating [18] through psychotherapy [19] and artistic creative processes [20], such as drama [21], music [22], and painting [23]. Art therapy has been considered a valuable way of expression, and art shows great hope in the future of sustainable development [24]. Recent research on the COVID-19 pandemic reports that art therapy supports the psychosocial needs of children and young adults [25]; prevents depressive symptoms [26]; relieves anxiety in hospitalized patients [27]; and promotes people’s physical, mental, and social health [28].

In the field of design, HTD refers to the use of design as an intervention to positively impact people’s physical and psychological well-being, prevent diseases, and improve health [29], reduce stress and anxiety, and improve patient satisfaction [30]. Healing and therapeutic architecture can help with exploring emotional and spiritual issues [31] and promoting mental health [16]. Healing and therapeutic landscape has been associated with improving individuals’ mental and physical health [32] and accelerating the recovery process of patients [33]. Healing and therapeutic space contributes to the well-being of patients [34] and relieves stress in an improved overall environment [35]. Additionally, it was argued that healing and therapeutic environment based on psychological stress theory and psychological development theory is a non-invasive method to reduce stress [36], which improves people’s quality of life, sense of freedom, anxiety level, sleep pattern, and weight loss [37]. Hence, considering the impact of the COVID-19 pandemic, using art, architecture, landscape, space, and the environment as a HTD medium can promote well-being and healthy lifestyles for all ages [38], and build inclusive, safe, resilient, and sustainable buildings [39] in line with the United Nations Sustainable Development Goals (SDGs), namely SDG 3 (Good Health and Well-Being) and SDG 11 (Sustainable Cities and Communities).

## 3. HTD and Blockchain (BC) Enhanced Information Modeling (IM)

With the advent of the digital age, the application of intelligent IM, such as BC, building information modeling (BIM), landscape information modeling (LIM), and city information modeling (CIM), to promote sustainable development has shown potential to promote health in different fields. BC is a decentralized trading and data management technology, which allows digital information to be distributed without copying or modifying [40,41]. A tracking system established for the COVID-19 pandemic can be further augmented using artificial intelligence and BC technologies [42]. The application of BC for health data, health information technology, and health care related studies promote the development of precision medicine [43] and improve health care services through shared distributed health data views [44]. Moreover, the application of BIM has embodied a new paradigm of environmental sustainability in the design concept of ‘healthy buildings’ [45]. BIM in health monitoring of building structures [46] eventually facilitates the health and well-being of building occupants [47]. With the rapid development of BIM, many studies explored the adaptability of IM in the field of landscape, and proposed the concept of LIM related to the discipline of landscape architecture [48]. Landscape design improves many climate and health issues [49]. It uses LIM for visualization [50] to help create sustainable cities with using fewer resources to mitigate environmental impacts [51]. Furthermore, applying the principles of BIM at a city level to build a CIM can serve as an analyzing and planning tool for future sustainable cities [52]. When implementing sustainability concepts in cities, CIM is used to improve public services and life quality for their citizens [53].

The integration of BC and BIM in information management during the life cycle of buildings brings two main benefits: (1) the transition process from BIM to BC will be easier and more efficient; and (2) data security can also be ensured during the construction phase of real estate [54]. Moreover, BIM enhanced by BC improves the security of information system across the whole life cycle of a building [55,56,57]. BC also has the potential to solve problems and challenges in the integration of LIM and CIM. BC, BIM, LIM, and CIM are complementary, and their integration can overcome their own defects and produce more advantages. Thus, the IM of BIM, LIM, and CIM driven by BC enhance the sustainable development in architecture, landscape, and city, which could be used as auxiliary tools to promote the attention to health problems, and improve the sustainability of architecture, landscape, and city design throughout the whole life cycle of design via applications of digital health.

In the face of the impact of the COVID-19 epidemic, it is necessary to think about how to effectively respond to the reality of global public health emergencies through design. While COVID-19 has created health challenges, it has also brought unprecedented opportunities for innovation [58]. With the development of science and technology, digital technology has stimulated the potential of design innovation, changed traditional design approaches [59], and enhanced the healing and therapeutic effect of art [60]. The HTD medias, such as art, architecture, landscape, space, and environment, and the BC enhanced IM shows great potential in promoting health. In general, art therapy and HTD not only improve health, but also have potential association to achieve sustainable development. Liu et al. (2021) argue that the application of HTD in the fields of architecture, landscape, space, and environment facilitates the achievement of the SDGs, and lays the foundation for the integration of BC and IM in the design process to drive the promotion of HTD as a healthy and well-being enabler [10].

## 4. Method

This paper adopts a mixed quantitative and qualitative method of literature analysis to develop the conceptual BC-HTD framework, which was validated via a pre-interview questionnaire survey and follow-up interviews with industry experts and academics. Using the quantitative bibliometric method, i.e., the Network Visualization of keyword co-occurrence generated by VOSviewer version 1.6.15, which has been developed by Nees Jan van Eck and Ludo Waltman at Leiden University’s Centre for Science and Technology Studies (CWTS) in the Netherlands, can reflect the relationship between different keywords, the frequency of occurrence, and different clusters [61,62]. Therefore, this paper conducts quantitative analysis based on the visual keyword co-occurrence network graph via VOSviewer software, which firstly analyzes keywords connections between BC, HTD, art therapy, and COVID-19, and then discusses the keywords connections between BC, BIM, and design. In qualitative literature content analysis, this paper analyzes the Double Diamond model of the British Design Council [63] as the classic design process of HTD. As among the various human-centered design thinking models, the Double Diamond model [64] has been regarded as one of the most efficient and persuasive design thinking process models since it was proposed by the UK Design Council in 2005 [65], which facilitates with discovering problems and finding solutions through the ‘vergence’ and ‘convergence’ thinking in the four design stages, i.e., Discover, Define, Develop, and Deliver [66]. Based on the design process of the Double Diamond model, the conceptual BC-HTD framework has been developed to address challenges during the HTD process one by one in the four design stages, which has been further validated by industry experts and academics at the “2021 China Society of Industrial and Applied Mathematics Blockchain Technology and Application Summit Forum” (CSIAM-BTAF 2021) [67]. The validation was conducted with five participants through pre-interview questionnaire survey and follow-up interviews. The participants are asked to use a score of 1–4 (1 = strongly disagree, 2 = disagree, 3 = agree, 4 = strongly agree) to assess the structural clarity of the conceptual framework, the appropriateness of the content, and the clarity of the process, which are used as the evaluation criteria to verify the conceptual BC-HTD framework based on the average score of all the participants.

**Figure 1:**
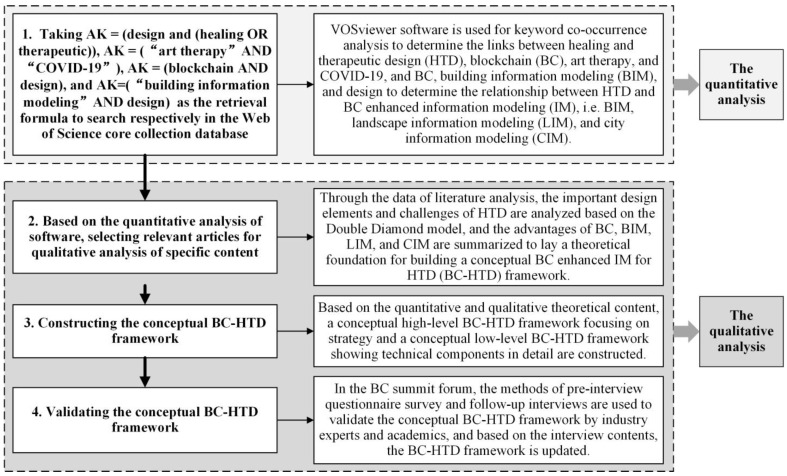
The flow chart of the research methodology.

As shown in Figure 1, the method flow chart in this paper is divided into four parts: (1) using quantitative bibliometrics analysis via VOSviewer software to analyze the keyword co-occurrence relationship between BC, HTD, art therapy, and COVID-19; and BC, BIM, and design to determine the relationship between HTD and BC enhanced IM; (2) based on the results of quantitative bibliometric analysis, selecting relevant articles for qualitative analysis of specific content, and laying a theoretical foundation that underpins the conceptual BC-HTD framework in the field of digital health; (3) constructing the conceptual BC-HTD framework based on the quantitative and qualitative analysis; and (4) validating the conceptual BC-HTD framework.

## 5. Results

### 5.1. Quantitative Analysis Results

**Figure 2:**
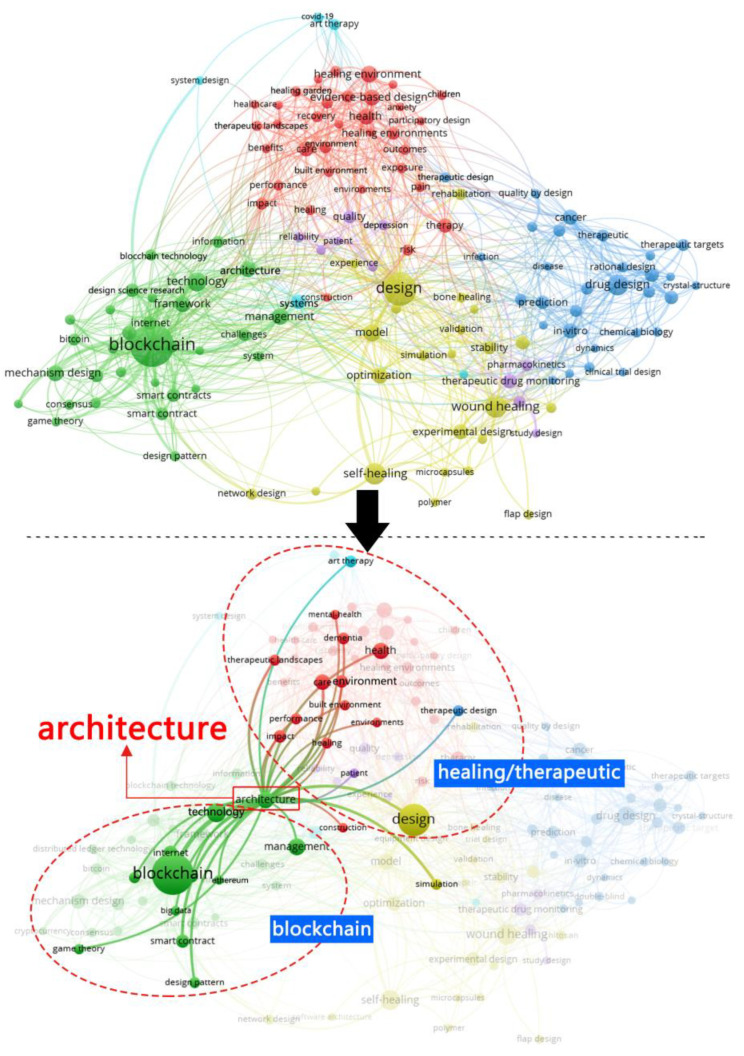
The relationship between healing and therapeutic design (HTD) and blockchain (BC) via VOSviewer software (generated by authors).

**Figure 3:**
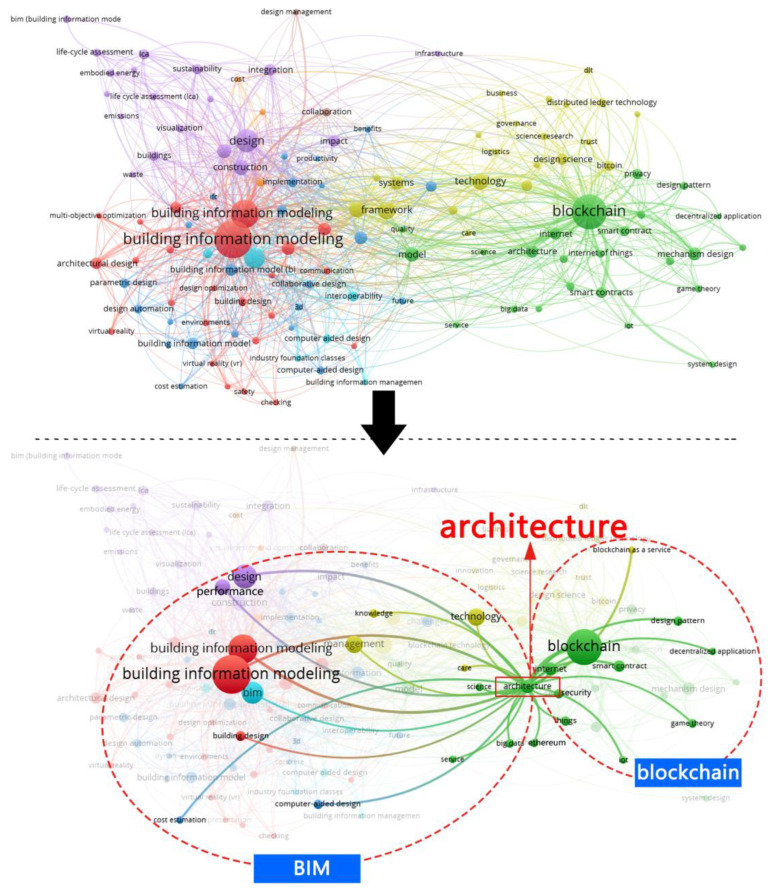
The relationship between BC, building information modeling, and design via VOSviewer software (generated by authors).

It can be seen from Figure 2 that the themes centered on HTD are mainly related to ‘healing environment’, ‘therapeutic landscapes’, ‘healing garden’, ‘built environment’, and ‘architecture’; and the themes centered on BC are mainly related to ‘architecture’, ‘information’, ‘management’, ‘framework’, and ‘smart contract’. In Figure 3, the themes on BC are associated with ‘smart contract’, ‘big data’, ‘internet’, ‘architecture’, and ‘sustainable design’; and the themes centered on BIM are mainly related to ‘building design’, ‘architecture’, ‘big data’, ‘internet’, and ‘environment’. Interestingly, both Figure 2 and Figure 3 uncover that ‘architecture’ has been utilized as a bridge between HTD and BC; BC and BIM, respectively. This echoes the potential connection between HTD and BC enhanced IM, and also indicates the possibility of the integration of BC integrating and BIM, LIM, and CIM to solve the challenges in the HTD process.

### 5.2. Qualitative Analysis Results

Based on the quantitative analysis results of visual keywords by the VOSviewer software, the following studies are selected for qualitative analysis of specific content. First, the important design elements of HTD and the challenges in the design process are analyzed from the design point of view. Then, the advantages of BC and IM are summarized from the design perspective.

#### 5.2.1. The Important Design Elements and Challenges of HTD

The HTD studies are mainly associated with the healing and therapeutic architecture [31,68,69], spaces [70,71,72], environment [36,73,74], and healing and therapeutic landscape design based on gardens [33,75,76]. In addition, HTD studies are focused on dementia patients to improve their environments and their quality of life [77,78]. Based on the application field of HTD, the healing and therapeutic architectural design is largely associated with BIM; and the healing and therapeutic landscape design corresponds to LIM. Furthermore, the design fields of comprehensive healing and therapeutic architecture, landscape, space, and environment indicate a strong link with CIM. BC has also been linked to LIM and CIM, to enhance HTD.

The HTD emphasizes important design elements in the presentation of architecture, landscape, space, and environment in promoting health via two means:

(1) Understanding and differentiating between different design variables. The change of design variables and the action mechanism of health impact can facilitate the individual and collective reflection of design variables in the overall design suggestions [68];
(2) Design decision-making process based on different forms of knowledge of stakeholders. Transforming the implicit knowledge of stakeholders into explicit knowledge in the design-driven process of healing and therapeutic architecture enhances communication among stakeholders, and avoids arbitrary decision-making to facilitate the design of healing and therapeutic architecture [79]. Additionally, healing and therapeutic designers need to make correct design decisions based on evaluations from the stakeholders, such as architects, patients, and healthcare providers [80]. Furthermore, the stakeholders’ judgments and decisions on the design of the healing and therapeutic built environment must be based on reliable evidence and knowledge [81].

Although HTD can promote human health through the medium of art, architecture, landscape, space, and environment, it also faces the following five challenges in the design process, such as hospital design:

(i) The changes and conflicts in design requirements. For example, due to the lack of consensus between hospital space design and nursing mode, it is easy to have conflicting design needs with nursing mode when designing the healing and therapeutic landscape of the hospital. It takes a long time to plan the design of the hospital from the perspective of architectural environment, which is difficult to flexibly respond to the changing needs over time [13].
(ii) Inconsistent design research framework and methodology. Although there are many studies on the healing and therapeutic environment with diverse methodologies, there is a lack of a unified research framework, which makes further research difficult [82].
(iii) Incomplete consideration of design features. The interrelationships between various design features are easily overlooked when there is HTD for the built and physical environments in which people with dementia live [14]. In addition, there are many issues related to aging that are not adequately addressed in the HTD for the environment in which people with dementia live, and environmental factors have not been considered comprehensively when intervening in the environment [15].
(iv) Lack of design evaluation tools and frameworks. Facing the dynamic needs of residents for the new generation of buildings, there is a lack of more comprehensive and informed tools to evaluate the effectiveness of the design and use of healing and therapeutic buildings, as well as a framework to clarify people-centered issues [83].
(v) Incomplete collection of design data. When there is HTD through art in the medical environment that are characterized by a diversity of intervention measures and results, the data collection is not comprehensive, data results are difficult to synthesize, and the evaluation of design results becomes difficult [16]. In addition, it is difficult to determine in the field of medical care whether the healing and therapeutic physical and social environment design results effectively improve the quality of life of patients, which reflects that the data on the impact of design on treatment results are inconclusive [34,84].

**Figure 4:**
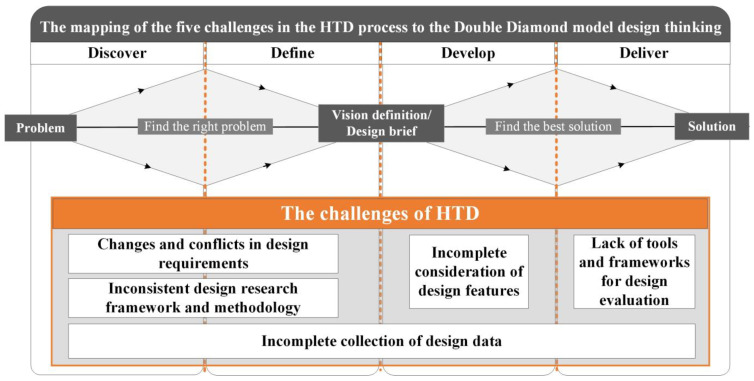
The mapping of the five challenges in the HTD process to the Double Diamond model design thinking (generated by authors).

In order to further explore the challenges of HTD across all the design stages, this paper maps the five challenges during the HTD process one by one in the four design stages of Discover, Define, Develop, and Deliver in the Double Diamond model, as shown in Figure 4. In the Stages of Discover and Define, HTD is faced with the challenges of changes and conflicts in design requirements, and inconsistent design research frameworks and methodologies. In the Stage of Develop, HTD faces the challenge of incomplete consideration of design features. In the Stage of Deliver, HTD is challenged by the lack of tools and frameworks for design evaluation. Furthermore, HTD faces the challenge of incomplete collection of design data across all the design stages.

#### 5.2.2. The Advantages of BC

The BC technology, with its features of disintermediation, immutability, and trust, has become one of the most promising and potentially transformative technologies in many industries, which contributes to the SDGs of the United Nations and produces extensive changes in some established industries and practices [85]. BC has key properties of transparency, security, and traceability [55,86,87,88]. In addition, decentralization is the most prominent and essential feature of BC technology [89]. In a data-driven world, BC technology potentially brings benefits to individuals and organizations that lack information infrastructure or have no access to reliable integrated data [90]. Furthermore, using BC technology as a reliable and secure decentralized information system can potentially improve the sustainability of building assets by providing transparent and comprehensive information to all stakeholders at different life cycle stages of an asset [91]. To designers, BC technology advances the collaborative design process in a novel way of working [92].

In the fields of art and healthcare, BC technology embodies the advantages of promoting the art economy, protecting intellectual property (IP) rights, and enabling data sharing. First, since BC technology brings efficiency and transparency, and adds value to products and services in different fields, the art world has begun to utilize BC technology to promote the art economy [93]. Moreover, the development of BC technology and smart contracts provides artists with a form of digital copyright management and creates a new tradable digital asset; such a trend means that IP operates differently in digital culture [94]. In the field of health care, when the health care system needs to be patient-centered to connect different systems and improve the accuracy of electronic medical records, BC technology provides a stable framework for health care data with the functions of data sharing, health record management, and access control [95]. In terms of information sharing, BC technology solves the problem of information sharing in the supply chain composed of the stakeholders, and the information sharing supported by BC can add value to strengthen the collaborative work of different types of supply chains, such as health and medical treatment, construction, and smart city [96].

In the field of information technology, BC is a shared data or information database that is unfalsifiable, untraceable, open and transparent, and collectively maintained [97]. Therefore, the adoption of BC technology can make the development of communities and enterprises more transparent, efficient and scalable, which accelerates the model of sustainable development [98]. In addition, BC technology, as an immutable digital record tool, allows different people and organizations to collaborate and create shared value, which solves increasingly complex problems in global value chains in pursuit of sustainable development [99,100]. Facing the three-tier new value system of value production, value recording, and value realization [101], BC provides democratized information access opportunities for the stakeholders through a new form of value delivery architecture [102]. Furthermore, BC has the following contributions in creating public value and promoting sustainable development: (1) building an authorization environment; (2) helping the design process to produce innovative solutions; (3) improving operation capacity; and (4) legalizing the final results [103]. In general, there are a number of advantages of BC technology, such as decentralization, openness, transparency, collective maintenance, and efficient sharing of databases, which lay the groundwork for facilitating the HTD process.

#### 5.2.3. The Advantages of Building Information Modeling (BIM), Landscape Information Modeling (LIM), and City Information Modeling (CIM)

In architectural design, BIM is a digital technology that uses a three-dimensional (3D) model to establish a set of project databases, which aid to achieve the SDGs of the project’s entire life cycle through the retrieval and application of information [104]. Based on the sustainable and transparent BIM collaborative work environment, the information of components can be copied and verified in the environment, so that all the participants communicate in time across the whole life cycle of the construction project, and every participant conducts scientific analysis and makes corresponding decisions through the design information shared by different design professionals [105]. Designers use BIM for visual design that intuitively helps with understanding the space of key parts and arranging corresponding parts reasonably, so as to make better use of limited space and effectively improve design efficiency and quality [106]. Moreover, the use of innovative BIM technology facilitates with realizing end-to-end communication, data exchange and information sharing among collaborators, and improving the cooperation among stakeholders [107]. The application of BIM in architectural design has the potential to improve the quality of information for key design decisions, and the visual technology provided by BIM helps designers to predict changes in the construction industry [108]. Furthermore, the BIM automatically associates with updating the design information in the healing building to avoid manual insertion that takes a lot of time [109]. As such, the BIM can be used as a shared knowledge resource of equipment information to ensure the integrity and accuracy of information and data exchange in the design process [110].

In addition, the rapid development of BIM technology provides a mature foundation for the development of LIM that is related to the design of landscape architecture, which expands the application objects of BIM. The difference between LIM and BIM is that the LIM has the core elements of terrain, vegetation, water, and the environment [111]. Based on accessible data stored in LIM for design creation, realization and management phases, landscape designers can be enhanced in decision-making and presentation of design outcomes, where reliable exchange of information increases efficiency, reduces errors, and minimizes the risk of corrections [112]. In addition, the visual design process provided by LIM improves participation and cognitive response of the stakeholders [113]. Furthermore, the use of LIM technology brings obvious benefits to the design process: (1) standardizing the knowledge of landscape design; (2) supporting the design information of different participants; and (3) improving information exchange between landscape, architecture, and urban design [114].

Furthermore, with the rapid development of BIM technology in cities, CIM is also flourishing in city research. The CIM is a technology based on BIM, geographic information system, and internet of things, which can realize the co-growth of digital cities and physical cities, and is also a necessary way to realize smart cities [115]. Integrating geographic information system and BIM systems to create CIM optimizes the management and monitoring of urban maintenance works and makes more efficient use of the allocated economic resources [116]. Moreover, the CIM is a digital 3D model of a city, which includes different types of information data [117]. The application of CIM technology helps to improve information management processes and control, and understand data in different domains [118]. Using CIM to build a visual 3D dynamic maintenance management platform brings the benefits of less intervention, high efficiency, and high precision in the new concept of ‘Community intelligent modeling’ [119]. By connecting BIM and CIM, users can be provided with an interactive and rich 3D city model environment in which data can be accessed, analyzed, and shared anytime and anywhere [120]. Furthermore, the CIM is an emerging research field [121], which adds value to urban space construction and its management [122]. Hence, the latest progress of BIM research plays a key role in the application of CIM [123].

In general, since the CIM includes the information of natural landscape, architecture, and infrastructure [124], BIM technology is the basis of LIM technology, and the LIM is the bridge between BIM and CIM, which integrates the design between city and architecture. The advantages of BIM, LIM, and CIM mainly include: (1) strengthening the communication and coordination of design work; (2) visualizing 3D design model of architecture, landscape, and city; (3) verifying the design results with the simulation of 3D model; (4) sharing common 3D models and coordination information; and (5) standardizing the design knowledge of architecture, landscape, and the city.

## 6. The Development of Conceptual BC Enhanced IM for HTD (BC-HTD) Framework

### 6.1. The Process of HTD Enhanced by BC

**Figure 5:**
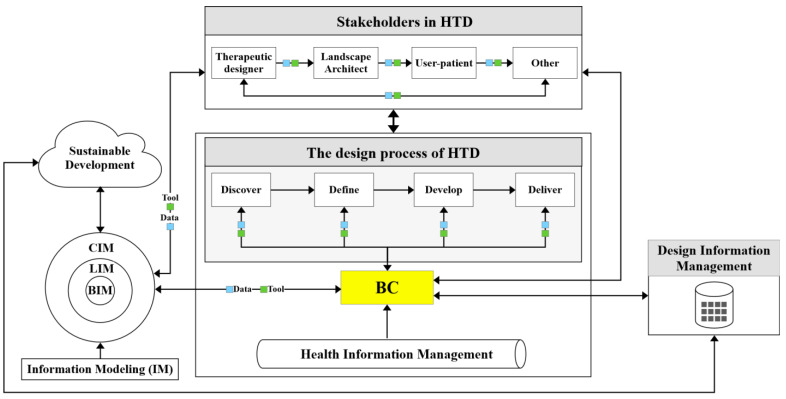
The process of HTD enhanced by BC (generated by authors).

As shown in Figure 5, the exchange of information within the context of sustainable development between stakeholders during HTD will be linked through IM driven by BC, which assists to understand and differentiate between different design variables. In terms of information flow, BC integrates data for health information management, as well as data and tools for visualizing 3D models provided by BIM, LIM, and CIM. Subsequently, the corresponding design data and tools driven by BC are obtained in the four design stages of Discover, Define, Develop, and Deliver within the Double Diamond design process. Additionally, the information in the design process is fed back to the design information management system via the BC technology—thus forming an interlocking and progressive relationship, and constantly enriching the data content of the design information management process. Finally, the management of HTD information for SDGs 3 and 11 to promote health and well-being and sustainable living can be realized.

### 6.2. The Knowledge Management for HTD Driven by BC

**Figure 6:**
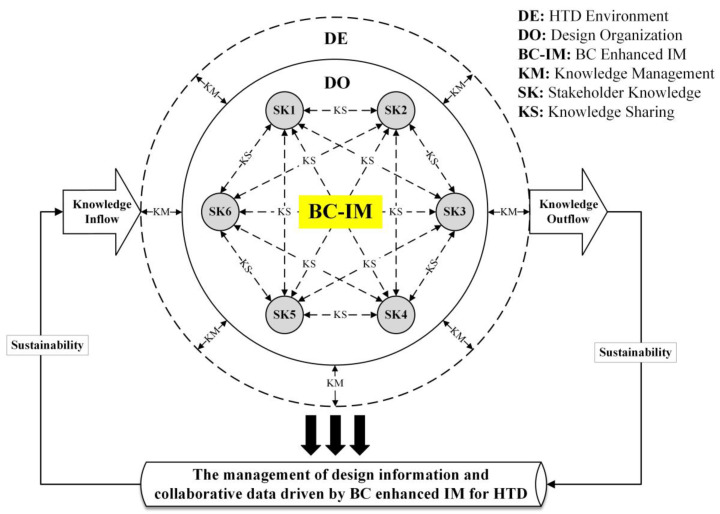
The knowledge management of HTD driven by BC (generated by authors).

In the process of BC-driven HTD, the knowledge management of the HTD, such as design information and health information, can inform the knowledge utilization process between the environment, organizations, and individuals from the inflow and outflow of knowledge, as shown in Figure 6. In the whole process of knowledge flow, the IM enhanced by BC is used to record and store the value of the HTD knowledge in art, architecture, landscape, space, and environment, which forms a set of available knowledge systems to achieve relevant indicators of sustainable development, and improves the generation and utilization of knowledge life cycle across the whole knowledge management stages. In addition, the IM driven by BC is used to transform tacit knowledge achievements of stakeholders into explicit knowledge to solve the accumulation, security, inheritance, repetition, sharing, and management problems of the HTD knowledge. Hence, the knowledge management of HTD through innovation driven digital transformation enables design decision-makers to carry out the decision-making process based on various forms of knowledge of stakeholders, which aids in realizing sustainable development in terms of knowledge flow.

### 6.3. The Potential Role of BC Enhanced IM in Addressing HTD Challenges

The potential roles of BC to enhance IM in line with the five identified challenges of HTD are:

(1) Responding flexibly to changes and conflicts in design requirements. Based on the advantages of BC in Section 5.2.2, the traceability database network of BC is used to summarize common information in healthcare, such as hospital design, and collaborative information for stakeholders in different time periods, which increases the consensus of design research and reflects the changing needs of users over time. For example, Wan et al. find that BC uses trusted ledgers to identify and understand information communicated between different stakeholders to achieve information consensus [96]. In addition, based on the advantages of BIM, LIM, and CIM in Section 5.2.3, the IM can gather the existing knowledge and information in art, architecture, landscape, and other related fields, strengthen the communication and coordination among stakeholders, and promote the collaborative design process.
(2) Unified design research framework and methodology. The results in Section 5.2.2 and Section 5.2.3 indicate that the traceable information database of BC enhanced IM facilitates with reviewing the past HTD cases, in which the most commonly used and most effective research methodology can be understood by sharing the common architectural, landscape, and city information.
(3) Fully considering the design characteristics. The BC enhanced IM directly transmits and uses design information collaboratively, and fully considers the interrelationship between various design features of the virtual built environment and physical environment. The IM presents the virtual 3D model to communicate design concepts and improve the quality of HTD. For instance, Lee et al. point out that digital manufacturing based on BIM provides instant design information and increases work efficiency, which avoids the problem of incomplete consideration of design characteristics caused by traditional paper documents [106].
(4) Improving design evaluation tools. The 3D models presented by the IM are used to simulate and verify the design results, and plan and analyze the elements of sustainable city development. Based on dynamic needs, the IM is facilitated to comprehensively and objectively evaluate the effectiveness of the design and the use of healing and therapeutic buildings, and clarify the human-centered problem framework. Furthermore, the consensus mechanism of the BC aids with design evaluation results. For example, Dantas et al. suggest that BIM and CIM can help city managers make assessments and decisions with simple and accurate data [53].
(5) Comprehensive collection of design data. BIM, LIM, and CIM enhanced by BC can bring seven main benefits to data collection: (a) BC provides transparent and comprehensive information to all the stakeholders at different stages of the HTD life cycle to improve design sustainability. For instance, Shojaei et al. show that BC stores and disseminates data information in different stages of asset construction, such as collection, verification, and extraction, which assists with achieving asset sustainability [91]. (b) BC establishes an open, transparent, collective maintenance, comprehensive and efficient design sharing information and database. (c) Design information for past, present, and future traceability is established through the traceability database network of BC. (d) The IM enables end-to-end communication, data exchange, and information sharing among stakeholders. (e) In the 3D models provided by the IM, accessing, analyzing, and sharing the data anytime and anywhere standardize the design knowledge and strengthen the information exchange.

**Figure 7:**
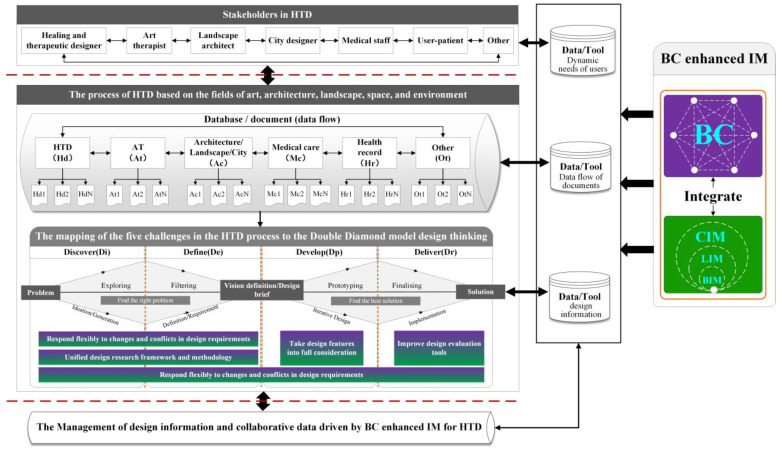
The potential roles of BC enhanced information modeling (IM) in addressing HTD challenges (generated by authors).

Figure 7 illustrates the potential role of data and tools provided by BC enhanced IM in stakeholders and design process. From the user’s point of view, BC enhanced IM facilitates the communication between the stakeholders during the HTD and gathers the dynamic needs of users through the traceability database network. From the perspective of design, the IM enhanced by BC establishes document data flow within the HTD process based on the domains of art, architecture, landscape, space, and environment, and uses design information and design tools throughout the four design stages of Discover, Define, Develop, and Deliver of the Double Diamond design thinking to solve the design challenges in the HTD process. As such, building design information management and collaborative data with BC enhanced IM for HTD strengthen the connection of HTD stakeholders and digitally drive the design process.

### 6.4. The Conceptual BC-HTD Framework for Sustainable Development

**Figure 8:**
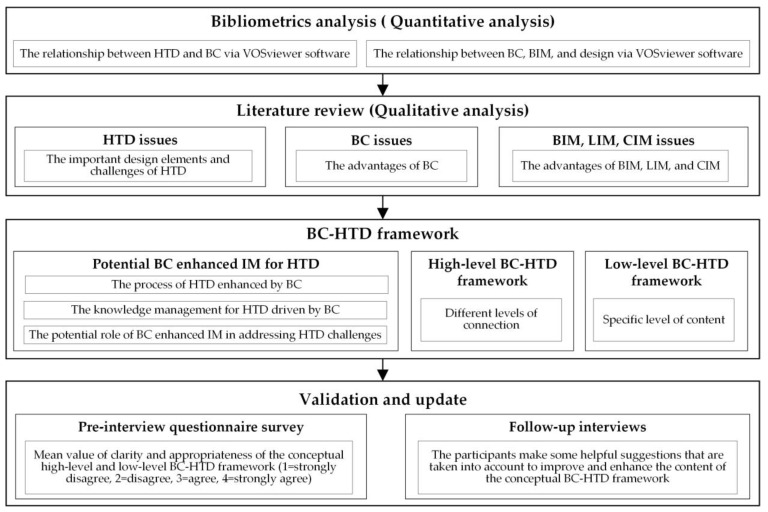
The conceptual BC-HTD framework design and development flow chart (generated by authors).

The development of the conceptual BC-HTD framework is based on the findings of the quantitative and qualitative analysis and the important design elements of HTD and associated challenges, as shown in Figure 8. The rationale of the conceptual BC-HTD framework is to use the BC enhanced IM, as tools to drive the design process during the HTD, and assist the flow of design knowledge and decision-making. The conceptual BC-HTD framework consists of a strategic high-level framework and a detailed low-level framework. The red coded numbers represent the important process actions of BC enhanced IM at different stages to identify the potential roles of the BC integrated IM (BC + BIM/LIM/CIM) in the whole life cycle of HTD, highlight the importance of design elements, and address HTD challenges.

#### 6.4.1. High-Level BC-HTD Framework

**Figure 9:**
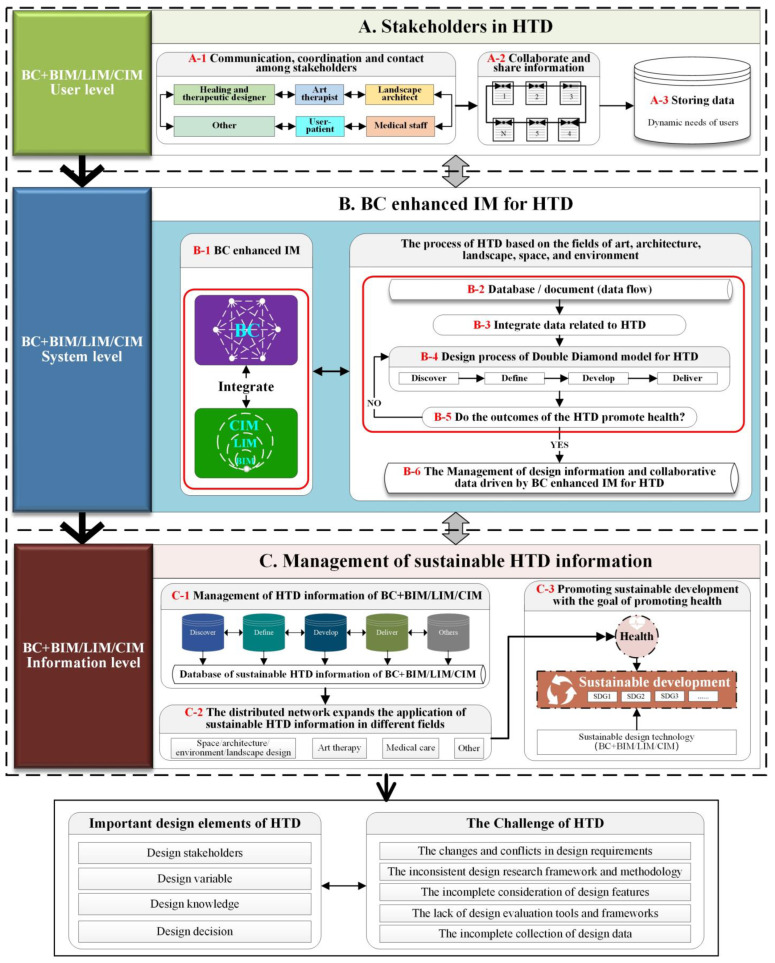
High-level BC-HTD framework (generated by authors).

Based on the relationship between the important design elements, HTD challenges, and the advantages of the BC enhanced IM, the conceptual high-level BC-HTD framework encompasses three levels comprising user, system, and information, as shown in Figure 9.

In the user level, BC + BIM/LIM/CIM consolidates the relationship between stakeholders of HTD; emphasizes information exchange, communication and coordination among stakeholders; and stores data of users’ dynamic needs. At the system level, the advantages of BC, such as openness, transparency, collective maintenance, comprehensive and efficient sharing of database, and traceability, address HTD challenges across the four stages of Discover, Define, Develop, and Deliver of the Double Diamond design process model. Additionally, the advantages of communication, collaboration, coordination, and visual 3D model of the IM assist the whole HTD process based on the fields of art, architecture, landscape, space, and environment. In the information level, BC + BIM/LIM/CIM enable the interaction between the user and the system to be transmitted to the design information level and interacting within the level. Moreover, the process totally contributes the HTD information to facilitate with achieving sustainable development via research framework, design requirements, design characteristics, evaluation framework, and design data. As such, design driving and design decision-making process are carried out based on the HTD information. In addition, the distributed network of BC + BIM/LIM/CIM expands the application of sustainable HTD information in different fields, such as art therapy, healthcare, architectural design, and landscape design, in which the BC + BIM/LIM/CIM is used as sustainable technology to promote sustainable development aimed at promoting health and well-being.

#### 6.4.2. Low-Level BC-HTD Framework

**Figure 10:**
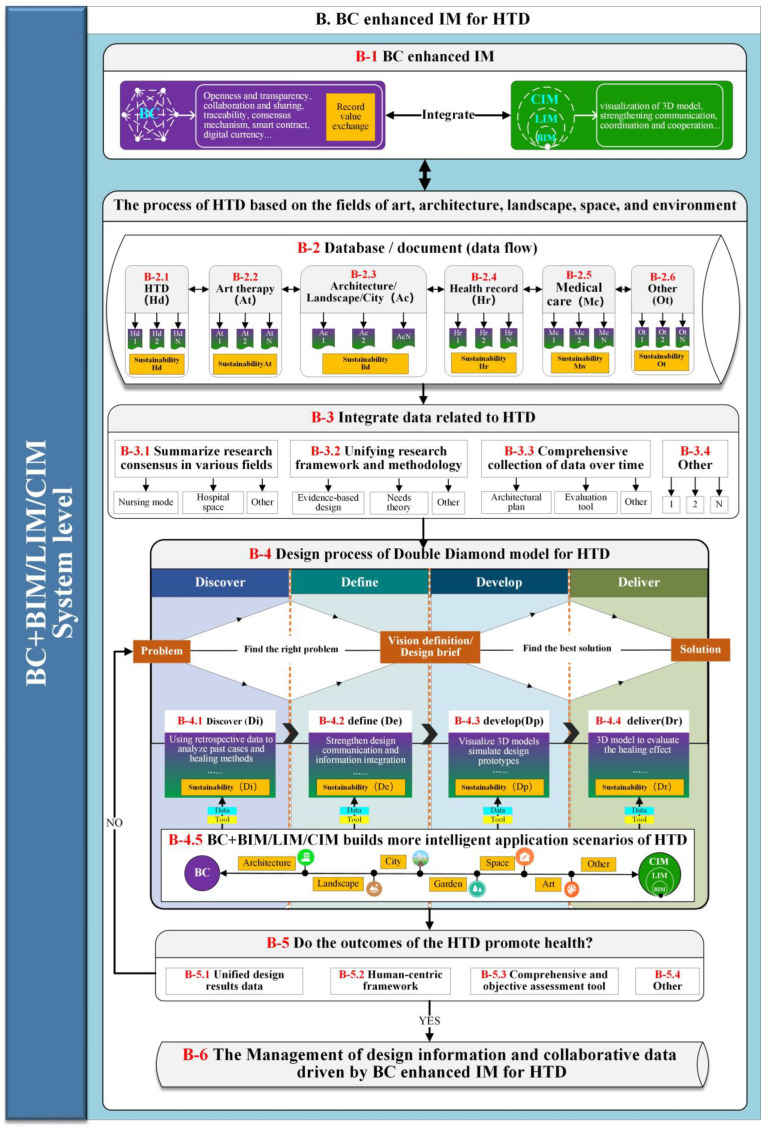
Low-level BC-HTD framework (generated by authors).

A conceptual low-level BC-HTD framework is shown in Figure 10. At the system level of the HTD, the advantages of the BC enhanced IM (B-1) highlighted in the design processes of HTD. In particular, the B-2 at system level reflects the databases/documents of HTD, art therapy, architectural/landscape/city design, health records, and medical care integrated before the design process of the Double Diamond model of HTD. These data at B-3 related to HTD summarize the research consensus in various fields, unify the research framework and methodology, and comprehensively collect the data over time. In addition, the B-4 at a system level promotes the potential role of data and tools in realizing the sustainability throughout the four design stages of Discover, Define, Develop, and Define with an intelligent application scenario based on the BC + BIM/LIM/CIM. Through the flow and use of data, the B-5 at the system level is to judge whether the outcomes of HTD promote health by unifying data on design outcomes, a human-centered framework, and comprehensive and objective assessment tools. If the outcome of the HTD promotes health, the design information and collaborative data used will be managed in the BC enhanced IM at B-6. If the results of the HTD do not promote health, the relevant data of the HTD will be reintegrated and returned to the Double Diamond design process of the HTD.

## 7. Validation and Update of the Conceptual BC-HTD Framework

The conceptual BC-HTD framework for digital health, as illustrated in Figure 8 and Figure 9, was accepted and invited to present [125], and validated by industry experts and academics at the CSIAM-BTAF 2021 [67]. All the participants involved in the validation agreed that the conceptual BC-HTD framework is clear, as shown in Table 1. In addition, the participants put forward their views to BC driven HTD based on the conceptual high-level and low-level BC-HTD frameworks, and pointed out that BC has a great potential in fully considering the important design elements of HTD and solving the challenges in the process of the HTD. Furthermore, the participants made some helpful suggestions that are taken into account to improve and enhance the content of the conceptual BC-HTD framework, which are:

(1) In the part of stakeholders, the BC can be used for multi-party trust connection, such as interconnection, mutual trust, and alliance chain.
(2) The approach of establishing incentives based on the BC encourages the stakeholders to fully contribute their own data.
(3) For the challenge of HTD, since the BC technology may not be able to work between multiple stakeholders for pure design behavior, the BC technology must have business collaboration, commercial behavior, and transaction behavior to cooperate.
(4) The pure function of the BC can be applied to the protection of IP rights. For example, the design draft and phased achievements of the designers can be recorded via the BC, which can be confirmed uniquely with digital assets on the BC technology to ensure non-infringement and measure the designer’s value by transaction behavior.
(5) The advantages of the BC, i.e., traceability, consensus mechanism, and smart contract, can help address the challenges of incomplete data collection during the HTD process.
(6) Building a real-time design knowledge base with BC enhanced IM is helpful to design decision-making.

**Figure 11:**
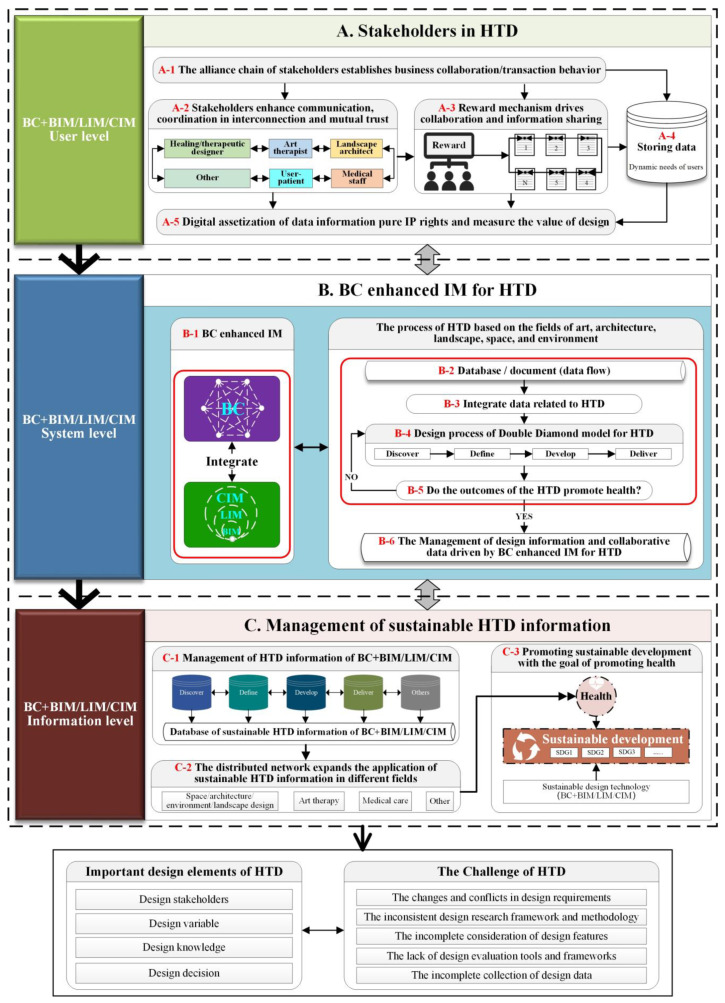
Updated high-level BC-HTD framework (generated by authors).

Furthermore, in line with the suggestions of the participants, the conceptual BC-HTD framework was updated on the user layer in the high-level BC-HTD framework, as shown in Figure 11, where the A1 was added in “The alliance chain of stakeholders establishes business collaboration/transaction behavior”, and A5 has been refined as “Digital assetization of data information pure IP rights and measure the value of design”. In addition, A2 was updated as “Stakeholders enhance communication and coordination in interconnection and mutual trust”, and the A3 is rewording as “Reward mechanism driven collaboration and information sharing”. Furthermore, the participants’ suggestions have been added to the user layer of the BC-HTD framework to establish business collaboration and transaction behaviors with stakeholder alliance chains, which ensure interconnection and mutual trust, encourage the stakeholders to contribute HTD data based on a reward mechanism, and quantify the design value of IP for digital assetization.

## 8. Discussion

### 8.1. Quantifying the Value of Design with BC Enhanced IM in HTD

The results in Section 5.2.2 indicate that the BC can assist to digitize IP and record value contributions in the process of information sharing. As such, BC has potential advantages in measuring the value of design. In the field of design, the value of designers cannot always simply be gauged from design works. This is because value is generated not only from suppliers (designers), but also from the experience services used by different participants and users throughout the whole design process [126]. Moreover, the concept of value in project management has changed from ‘value management’ to ‘understanding how the stakeholders evaluate the value of different things [127]. Therefore, when the value of designers cannot be measured by design works, the BC represents a huge advantage. Based on the decentralization, transparency, and security characteristics of BC, the BC is a good value management method, where the BC encapsulates qualitatively different data and improves management efficiency, effectiveness, and performance of participants [128]. For enterprises, the real value of the enterprise can be assessed using the automatic decision-making value system of the BC [129]. Furthermore, the BC serves as a record that effectively determines the value of participants’ contributions and functions as a true sharing economy [130]. As healing and therapeutic gardens play a key role in reducing the emotional impact of hospitalization in the field of health care, future research should quantify and monetize the therapeutic benefits of HTD [131].

**Figure 12:**
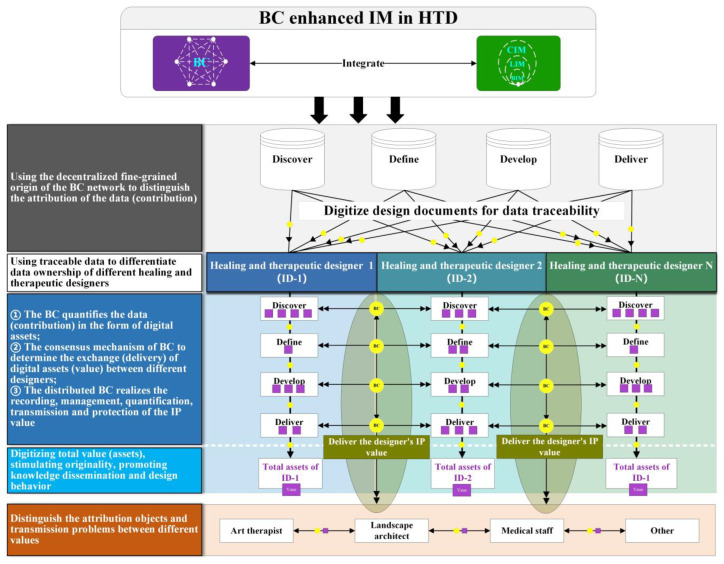
Quantifying the value of design with BC enhanced IM in HTD (generated by authors).

The BC tracks the contribution of network participants to measure the value brought with adequately fine granularity [132] and protect the corresponding IP rights based on the BC consensus mechanism contributed by participants [133]. Additionally, the IP value of each designer is recorded, managed, quantified, transmitted, and protected in the consensus mechanism of BC technology, which can stimulate the original motivation of designers, disseminate knowledge and drive the design behavior, as shown in Figure 12. In the HTD stages of Discover, Define, Develop, and Deliver, the decentralized fine-grained provenance tracking and consensus mechanism of BC are used to distinguish the attribution objects of data, realize the traceability of data, and convert the design documents into digital assets, which can record, manage, transmit, and quantify the IP value of designers. Thence, the future research could use BC to distinguish the ownership of data in different design stages and digitalize design information.

### 8.2. BC-Based Non-Fungible Token to Quantify the Value of Art Therapy

**Figure 13:**
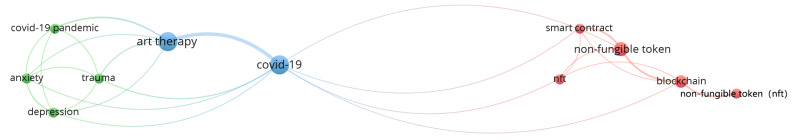
The relationship between art therapy and non-fungible tokens in the context of COVID-19 (generated by authors).

Moreover, the results of the quantitative analysis presented in Section 5.1 suggest that there is a certain connection between art therapy and BC, with architecture as a bridge. When using the artistic medium for HTD, the outcome of artworks, such as paintings and music, can represent their value to HTD. In the field of art, BC can facilitate solving the problem of transparency of artworks, and the use of new forms of non-fungible token (NFT) in the BC, which secures the rights of art works, allows precise control of copyright and ownership, and highlights new technologies addressing IP issues in digital art [134]. With analyzing the keyword co-occurrence relationship between art therapy and NFT in the context of COVID-19 in the Web of Science core collection database via VOSviewer, it can be seen that the COVID-19 is a bridge between art therapy and NFT, as shown in Figure 13, which indicates a potential association in measuring the value of art therapy in the way of NFT. When quantifying the value of an artwork, NFT can bring greater certainty to issues of ownership and authenticity of artwork [135]. In the application of digital assets, NFT has gained obvious public attention by recording the ownership of digital assets such as images, music, videos, and virtual works through smart contracts of the BC [136]. Based on the unique properties of BC’s distributed and immutable records, NFT creates a record of the source of digital artworks in the BC and operate the copyright management of artworks [137]. Additionally, each uniquely identifiable entity can be mapped to the NFT’s transfer by tying each entity to the BC [138]. Therefore, future research could use the approach of NFT in the BC to quantify the value of art-mediated healing and therapeutics, and drive the healing and therapeutic behavior of art therapists in the form of digital assets to promote people’s health and well-being.

Furthermore, BC presents a great potential in the tokenization of digital assets [139]. BC technology creates a trusted environment based on the essence of transparency, and makes information publicly used in the whole network in a trusted environment, which can maintain the integrity and invariance of data, promote stakeholders in different chains to jointly create value, ensure the availability of information, and provide a coordination mechanism [140]. Therefore, quantifying the value of design with BC enhanced IM drives HTD behavior and promotes achievement of SDGs. Interestingly, since new digital technologies contribute to the concept of the Metaverse in which resources connect the virtual digital world to the physical world [141], the properties of BC in the sharing economy creates sustainable business models in the Metaverse [142]. The future development trend of Health Metaverse is prospected in the way of quantifying the design value of BC enhanced IM including BIM, LIM, and CIM, and NFT management of digital art assets, which has certain social significance to achieve sustainable development from the field of digital health.

## 9. Conclusions

At present, the HTD, which promotes health to achieve sustainable development, faces design-related challenges in the fields of art, architecture, landscape, space, and the environment. The development of emerging IM, such as BIM, LIM, and CIM, is enhanced by BC to promote sustainable development, bringing immediate advantages in solving the challenges of HTD in the field of digital health. Therefore, this paper is the first attempt to apply the advantages of BC to IM with a view to enhancing the HTD process in line with the Double Diamond model of the British Design Council, considering important elements and challenges in the design process. From a research perspective, in the aftermath of the COVID-19 epidemic, this study investigates the application of integration of BC with BIM, LIM, and CIM in HTD, which is aimed at promoting health and well-being heading to the sustainable development. In terms of research content, this paper proposes a BC-driven HTD process and a management process of design knowledge. Moreover, the conceptual BC-HTD framework driven by BC + BIM/LIM/CIM has a high-level framework of three levels, i.e., user, system, and information, and a low-level framework of detailed content at the system level, which has been constructed using a mixed quantitative and qualitative method of literature analysis, and validated and updated via pre-interview questionnaire and follow-up interviews with industry experts and academics. This paper analyzes the process of BC enhanced HTD and the knowledge management of HTD to aid design decisions in managing design information. The potential of BC to quantify the HTD value is constantly being tapped. The results of this study can help future research to quantify the value of design in the form of NFT based on the extended advantages of BC in the field of design, which can fully mobilize the advantage potential of HTD to promote health, and realize the vision of Health Metaverse in future digital health. In addition, the form of NFT based on the BC facilitates mobilizing the healing and therapeutic behaviors of designers in the use of the advantage potential of HTD to promote health in achieving the SDG 3 (Good Health and Well-Being) and SDG 11 (Sustainable Cities and Communities). However, the quantitative analysis based on VOSviewer software in this paper focuses on the WOS core collection database. The conceptual BC-HTD framework is validated by industry experts and academics. As such, the follow-up research could conduct quantitative analysis of multiple databases, such as Scopus and ScienceDirect, and explore a practice approach for BC enhanced IM for HTD to promote health in the context of COVID-19 based on the conceptual BC-HTD framework.

## Figures and Tables

**Table 1:** Mean value of clarity and appropriateness of the conceptual high-level and low-level BC-HTD framework (responses of industry experts and academics).

Evaluation Criteria	High-Level	Low-Level
Clarity of the structure	3.70	3.50
Appropriateness of content	3.15	3.00
Clarity of flow	3.40	3.20

## Data Availability

Publicly available datasets were analyzed in this study. These data can be found here: https://login.webofknowledge.com/ (accessed on 30 April 2022).
